# Supplementary material for: Predictions of potential geographical distribution and quality of Schisandra sphenanthera under climate change
Source: PeerJ. 2016 Oct 20;4:e2554. doi: 10.7717/peerj.2554 (PMC5075693; doi:10.7717/peerj.2554)
Supplement: Supplemental Information 1 [file peerj-04-2554-s001.docx]

Fruits contents of Schisantherin A in sample of *Schisandra sphenanthera* (%)

| Sampling point code | Schisantherin A (%) | Sampling point code | Schisantherin A (%) |
| --- | --- | --- | --- |
| HN-XW007 | 0.638 | ZJ-LA009 | 0.279 |
| HN-XW009 | 0.868 | AH-JZ004 | 0.275 |
| HN-XW010 | 0.778 | AH-JZ005 | 0.32 |
| HN-XW011 | 0.443 | AH-JZ006 | 0.076 |
| HN-XW013 | 0.926 | AH-JZ007 | 0.08 |
| HN-LS001 | 0.657 | AH-JZ010 | 0.211 |
| HN-LS005 | 1.227 | SN-YPHB005 | 0.582 |
| HN-LS006 | 0.701 | SN-YPHB008 | 0.918 |
| HN-LS011 | 0.492 | SN-YPHB010 | 1.017 |
| HN-LS020 | 0.281 | SN-YPHB011 | 0.424 |
| SX—LC001 | 0.496 | SN-FZ004 | 1.012 |
| SX—LC004 | 0.579 | SN-FZ006 | 0.739 |
| SX—LC008 | 0.211 | SN-ZA001 | 1.071 |
| SX—LC011 | 0.452 | SN-ZA005 | 1.049 |
| GS—ZQ001 | 0.612 | SN-ZA006 | 1.049 |
| GS—ZQ002 | 0.114 | SN-ZA008 | 1.153 |
| GS—ZQ005 | 0.141 | SN-ZA014 | 0.512 |
| GS—ZQ009 | 0.469 | SN-XY006 | 0.526 |
| GS—ZQ014 | 0.249 | SN-XY009 | 1.443 |
| GS—ZQ018 | 0 | SN-XY014 | 0.71 |
| GS-HT001 | 0.876 | SN-XY015 | 0.072 |
| GS-HT004 | 0.501 | SN-XY018 | 0.015 |
| GS-HT005 | 0.175 | SN-FP005 | 0.961 |
| GS-HT007 | 0.468 | SN-FP007 | 0.194 |
| GS-HT008 | 0.262 | SN-FP009 | 0.174 |
| SC-QC002 | 0.361 | SN-FP012 | 0.66 |
| SC-QC006 | 0 | SN-FP016 | 0.506 |
| SC-QC009 | 0.184 | SN-LB005 | 0.92 |
| SC-QC0013 | 0.255 | SN-LB007 | 0.931 |
| SC-QC0014 | 0.155 | SN-LB010 | 0.381 |
| SC-QC0016 | 0 | SN-LB013 | 1.948 |
| CQ-WX003 | 0 | SN-LB014 | 0.468 |
| CQ-WX005 | 0.923 | SN-FX002 | 0.609 |
| CQ-WX006 | 0.11 | SN-FX004 | 0.19 |
| CQ-WX008 | 0.118 | SN-FX008 | 0.57 |
| CQ-WX011 | 0.394 | SN-FX010 | 0.261 |
| CQ-WX012 | 0.187 | SN-LX002 | 0.39 |
| ZJ-LA001 | 0.469 | SN-LX003 | 0.393 |
| ZJ-LA002 | 0.268 | SN-LX007 | 0.532 |
| ZJ-LA005 | 0.308 | SN-LX010 | 0.151 |
| ZJ-LA006 | 0.316 | SN-HX001 | 0.734 |
| SN-HX004 | 0.723 | SN-HX009 | 0.141 |
| SN-HX006 | 1.157 |  |  |
